# Individually Modeled 3D Printed Toothbrush and Interproximal Brush Handle With Name for Patients With Limited Manual Dexterity

**DOI:** 10.7759/cureus.27097

**Published:** 2022-07-21

**Authors:** Shreya Colvenkar, Ramesh Kunusoth, Rathod Prakash, Aditya Mohan Alwala, Sabavat Ashok Kumar

**Affiliations:** 1 Department of Prosthodontics, MNR Dental College and Hospital, Sangareddy, IND; 2 Department of Oral and Maxillofacial Surgery, MNR Dental College and Hospital, Sangareddy, IND; 3 Department of Prosthodontics, No. 8 Dental Unit Assam Rifles, Assam, IND

**Keywords:** interproximal brush, customized, oral hygiene, handle, toothbrush

## Abstract

Maintenance of proper oral hygiene is a cumbersome task when hand and finger movement is restricted. The customized handle allows better grip on the toothbrush, making it easier to brush teeth or dentures. This increases self-esteem by reducing their dependency on others. This article describes a method of fabricating a 3D printed two-in-one customized handle for a patient with limited manual dexterity. The 3D printed handle is simple and requires less fabrication time than other methods. The customized handle is strong, durable, and resistant to water absorption. It also allows reuse with a new brush design, thus saving additional time and cost. In addition, using a customized toothbrush handle is more effective in oral plaque control than a standard toothbrush.

## Introduction

Individuals with physical dysfunction like arthritis or stroke face a greater challenge in maintaining oral care than people who do not have disabilities. It is essential to maintain good oral hygiene as neglecting it causes dental diseases such as dental caries, gingivitis, and periodontitis [1]. Malnutrition is associated with poor oral hygiene. Nutrition intake is affected because of impaired sensory and masticatory function. Once dental diseases set in, it is difficult and costly to treat. Family members need to spend additional time for dental visits by taking time off from their work.

To maintain good oral health, adequate plaque control plays a crucial role. This can be achieved by brushing teeth using the correct technique [2,3]. Brushing can be done correctly when the toothbrush reaches every corner of the mouth. Brushing requires manual dexterity, which is mostly diminished in patients with arthritis or stroke. The limited movement of hand and finger makes holding the toothbrush a cumbersome task. So, it is essential to have a handle that fits correctly in one’s hand to carry out brushing correctly [4].

This article describes a method of making 3D-printed customized handles that are individually adapted for use by an elderly patient with limited manual dexterity. The customized handle allows better grip on the toothbrush, thus making it easier for them to brush their teeth or denture. The technique is simple, cheap, and requires less time than other techniques. The same handle can be used for toothbrushes and an interproximal toothbrush.

## Technical report

Receive the patient with a calm and caring attitude before the start of the procedure. Explain in detail the procedure to be carried out. Ask the patient to select the toothbrush of his/her choice. Lightly grease the toothbrush handle with Vaseline to easily remove the toothbrush from the handle. Mold a dense silicone putty impression material around the toothbrush handle. Ask the patient to hold the toothbrush in hand as if he/she is preparing to brush the teeth. Ask the patient to squeeze the material gently to get the proper shape of the hand. On completion of polymerization, remove the toothbrush from the molded handle.

**Figure 1:**
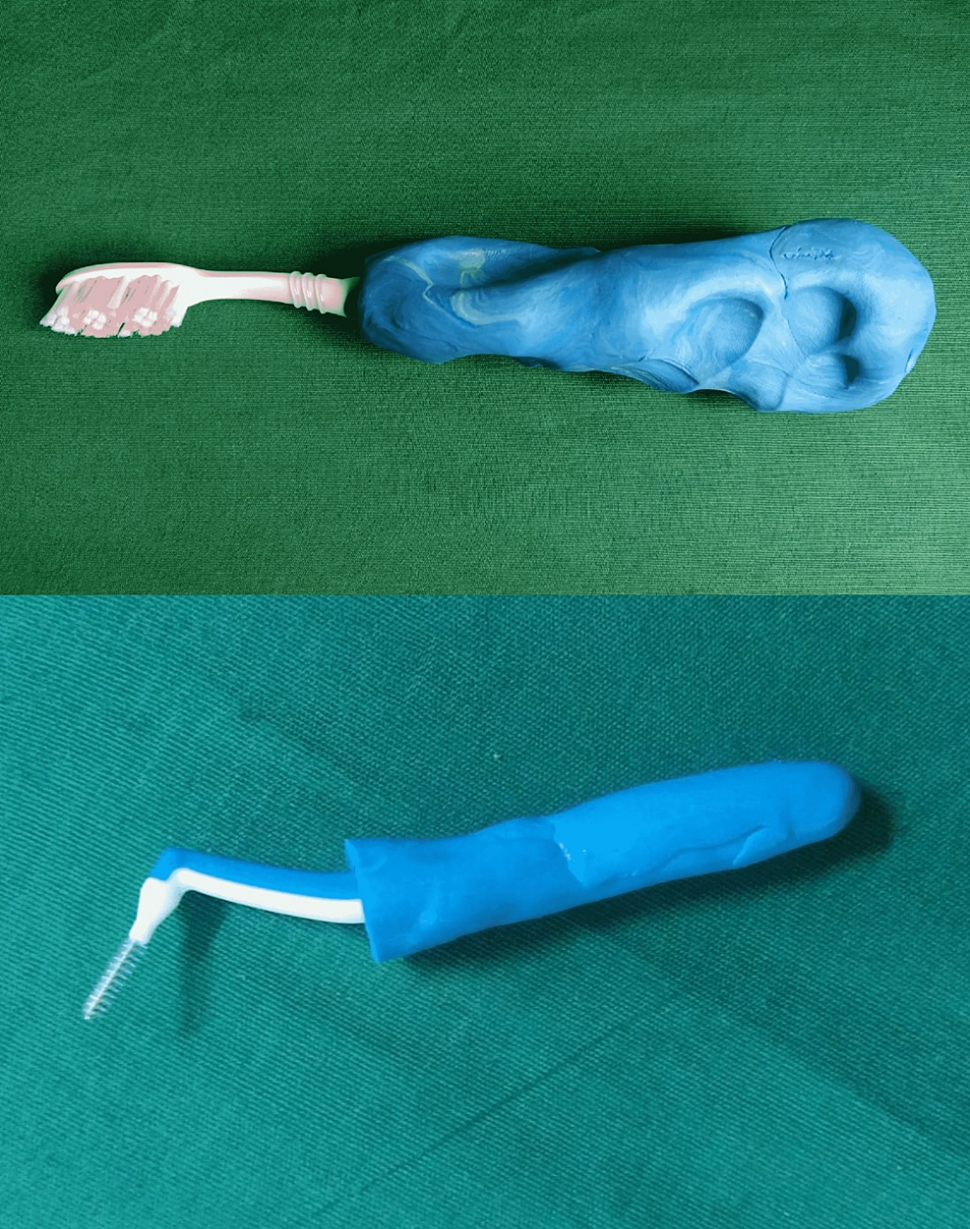
Molded handle around toothbrush and interproximal brush.

Select the interproximal brush of the patient’s choice. Apply separating medium to the inner surface of the molded handle and the interproximal brush. Mold a thin layer of dense silicone putty impression material around the interproximal toothbrush and insert it into the molded handle of the toothbrush (Figure 1).

**Figure 2:**
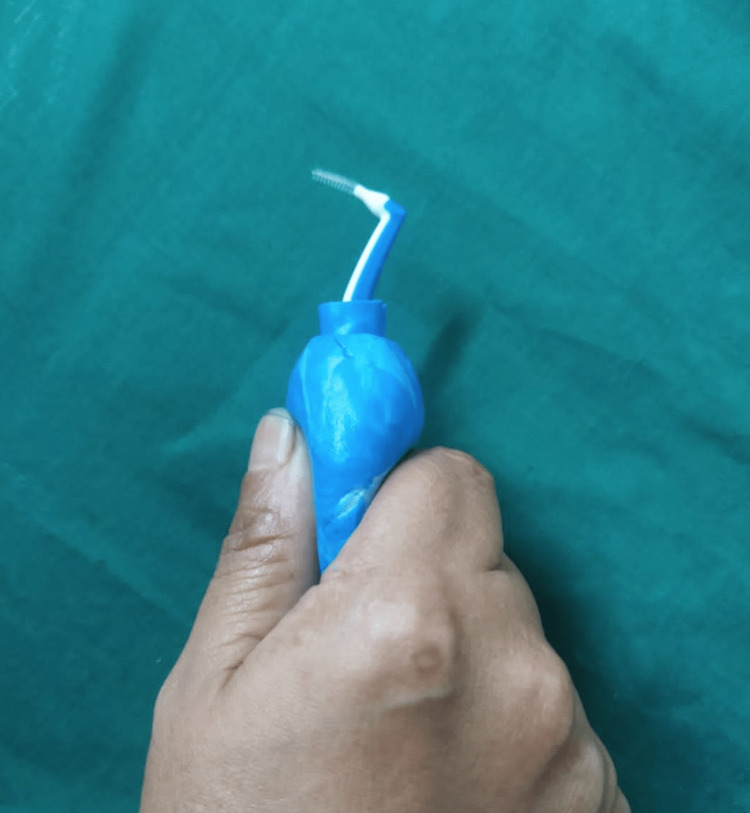
Correct fit of interproximal handle into toothbrush handle.

Separate it from that molded handle once the impression material sets. Separate the interproximal brush from the molded impression material. Insert the interproximal brush handle into the toothbrush handle to check the fit (Figure 2).

**Figure 3:**
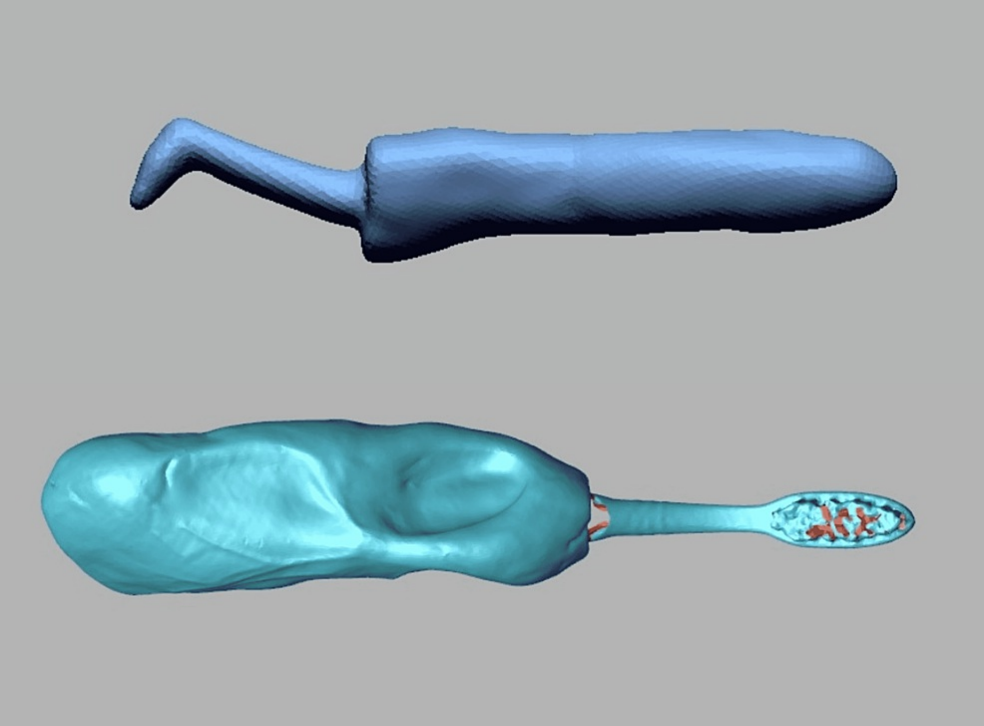
3D printed image.

Fabricate both the customized handle with 3D printing software Mimics (Materialise NV; Belgium) (Figure 3).

**Figure 4:**
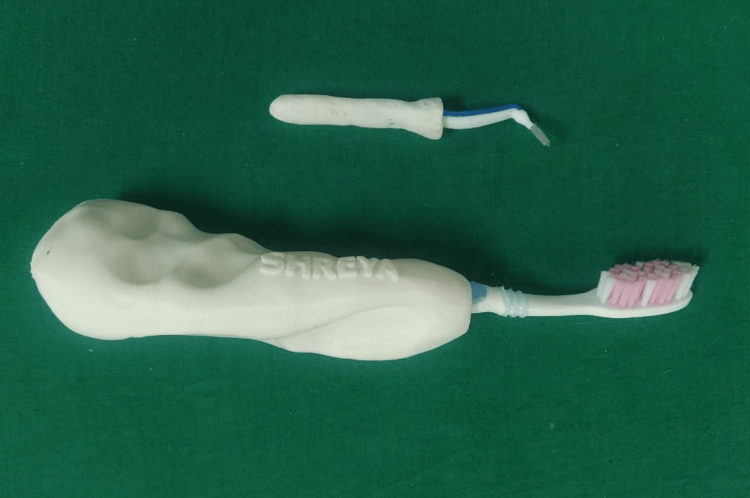
3D printed handle.

Print using polylactic acid material with a 3D printer Ultimaker 2+ (Ultimaker BV; North America) (Figure 4).

**Figure 5:**
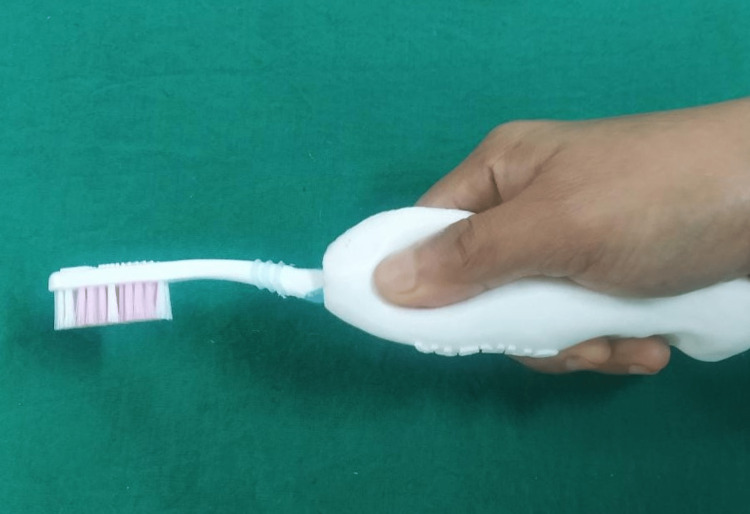
3D printed handle with toothbrush.

**Figure 6:**
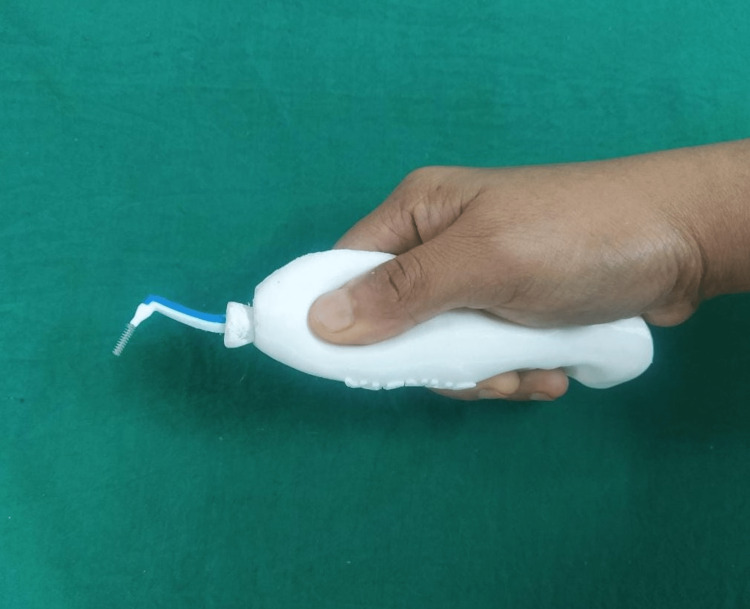
3D printed handle with interproximal brush.

Lastly, check the alignment of the toothbrush and interproximal brush (Figures 5-6).

Instruct the patient about the handling of the toothbrush and interproximal handles. Explain to the patient that once brushing is completed with a customized handle, insert an interproximal brush with its handle into the toothbrush handle to carry on with oral hygiene.

## Discussion

Plaque control is essential not only for oral health but also for general wellbeing. The various microorganisms present in plaque can act as a source of infection. These microorganisms may be aspirated, causing GI or respiratory problems like cases of pneumonia [5], especially in bedridden or hospitalized patients [4]. The easiest and most efficient method of removing biofilm is by brushing. In addition, dental flossing and chemical methods using antiplaque agents are more time-consuming and insufficient without brushing [6,7].

Limited manual dexterity will certainly hinder oral hygiene measures. However, this can be avoided by using a handle individually adapted to one’s own hand. This customized handle will increase the volume of the handle, making it easier to brush teeth. It will also reach all the mouth regions to clean effectively because of better control on the handle. An individual can brush on their own by reducing dependency on others. This freedom of self-brushing will increase their self-respect and mental health by reducing their reliance on caregivers.

Literature mentions a lot of modifications in toothbrush handles like handles with an elastic cuff, bicycle handlebar grip, and tennis ball handles. However, they are not customized to the patient’s hand. Individually adapted handles can be made using silicone putty [8], light cured composites [9], or acrylic resins [10]. Fabricating a handle with light‐polymerized composite material is simple and quick but at the price of high cost. Also, it needs to be repeated with a new toothbrush design. Furthermore, the silicone putty is not long-lasting because of its property to crack. Acrylic resins are readily available in a dental office, but laboratory procedure is cumbersome and lengthy.

This article describes a simple technique for fabricating a 3D printed handle with a patient’s name from polylactic acid material. Polylactic acid material is advantageous because of its low cost, high-dimensional accuracy, good surface finish, and strength. In 3D printing, various layers of material are added one by one to create a three-dimensional object with the help of computer software. This method will create highly accurate complex geometries in a minimum time of three hours with ease. The handle is made from durable material to be used for a long time and costs approximately 2500 rupees. The same handle with minor modifications can be reused with different toothbrush designs minimizing the time and cost of making a new one in the future. This two-in-one handle can also be used with an interproximal toothbrush.

To maintain good oral care, the focus should be on preventing oral diseases and reducing the need for comprehensive dental treatment. The final objective should focus on maintenance and self-care to improve quality of life. The use of an individually modeled 3D printed handle with the patient’s name will definitely increase self-esteem and undeniably contribute to a better quality of life because of better maintenance of oral hygiene and fewer dental visits. In addition, satisfactory oral hygiene will make nutritional intake possible, thus keeping the body healthy and strong.

## Conclusions

This article describes a method of making a two-in-one customized handle for an interproximal brush and toothbrush for a patient with limited manual dexterity. The technique is very simple and requires less time compared to other techniques. In addition, using an individually adapted 3D printed toothbrush handle is more effective in plaque control than a standard toothbrush.
